# A simulation study to evaluate the performance of five statistical monitoring methods when applied to different time-series components in the context of control programs for endemic diseases

**DOI:** 10.1371/journal.pone.0173099

**Published:** 2017-03-06

**Authors:** Ana Carolina Lopes Antunes, Dan Jensen, Tariq Halasa, Nils Toft

**Affiliations:** 1 Division for Diagnostics and Scientific Advice—Epidemiology, National Veterinary Institute–DTU, Bülowsvej 27, Frederiksberg C, Denmark; 2 Section for Production and Health, Department of Large Animal Science–KU, Grønnegårdsvej 8, Frederiksberg C, Denmark; Hokkaido University Graduate School of Medicine, JAPAN

## Abstract

Disease monitoring and surveillance play a crucial role in control and eradication programs, as it is important to track implemented strategies in order to reduce and/or eliminate a specific disease. The objectives of this study were to assess the performance of different statistical monitoring methods for endemic disease control program scenarios, and to explore what impact of variation (noise) in the data had on the performance of these monitoring methods. We simulated 16 different scenarios of changes in weekly sero-prevalence. The changes included different combinations of increases, decreases and constant sero-prevalence levels (referred as events). Two space-state models were used to model the time series, and different statistical monitoring methods (such as univariate process control algorithms–Shewart Control Chart, Tabular Cumulative Sums, and the V-mask- and monitoring of the trend component–based on 99% confidence intervals and the trend sign) were tested. Performance was evaluated based on the number of iterations in which an alarm was raised for a given week after the changes were introduced. Results revealed that the Shewhart Control Chart was better at detecting increases over decreases in sero-prevalence, whereas the opposite was observed for the Tabular Cumulative Sums. The trend-based methods detected the first event well, but performance was poorer when adapting to several consecutive events. The V-Mask method seemed to perform most consistently, and the impact of noise in the baseline was greater for the Shewhart Control Chart and Tabular Cumulative Sums than for the V-Mask and trend-based methods. The performance of the different statistical monitoring methods varied when monitoring increases and decreases in disease sero-prevalence. Combining two of more methods might improve the potential scope of surveillance systems, allowing them to fulfill different objectives due to their complementary advantages.

## Introduction

Surveillance and monitoring systems are critical for the timely and effective detection of changes in disease status. Over the last decade, several studies have applied different statistical monitoring methods for detecting outbreaks of (re-)emerging diseases in the context of syndromic surveillance in both human and veterinary medicine [1–3]. Different types of models (such as linear models, logistic regression and time-series models) have been implemented in the context of syndromic surveillance in order to evaluate the performance and implementation of these methods [4].

However, it may not be possible to make generalizations about the performance of these methods when used for monitoring endemic diseases and control programs. In this case, the availability of control measures (such as vaccination or health-management programs) results in lower incidence rates for endemic diseases than for (re)-emerging diseases. The dynamics of disease spread and immunity within a population from previous exposure also contribute to a lower incidence, resulting in slow and gradual changes in incidence and prevalence for endemic diseases [5]. It is important to follow-up on implemented control strategies in order to reduce and/or eliminate a specific disease [6]. Unexpected changes (such as an increase in disease prevalence or a failure to achieve a target value of disease prevalence within a certain period of time) indicate that the implemented strategies should be revised. When a control program fails to achieve certain goals, it can have a devastating impact on herds with susceptible animals.

In previous work, we assessed the performance of univariate process control algorithms (UPCA) in monitoring changes in the burden of endemic diseases based on sentinel surveillance [7]. However, these methods were not tested in the context of voluntary disease control and monitoring programs. In such cases, the frequency of testing depends on the monetary value of the animal and not just on the impact of the disease [6]. Programs for monitoring endemic diseases include the Danish Porcine Reproductive and Respiratory Syndrome Virus (PRRSV) monitoring program. Despite disease-control efforts, PRRSV has contributed to economic losses since its first diagnosis in 1992 [8]. Monitoring of PRRSV is primarily based on serological testing within the Specific Pathogen Free System (SPF System) [9]. The frequency of testing depends upon the health status of the herd within this system. As a consequence, the number of samples is not constant and it is necessary to use methods with a more dynamic structure, allowing the parameters to change over time, thus taking into account the variation in sample size. Previous studies have also discussed the influence of variation in the number of samples (i.e. the noise present in data) on the performance of different monitoring methods [7,10].

State-space models have a flexible structure, allowing parameters to be updated for each time step [11]. In addition, they can be decomposed, and changes in the components (such as trends and seasonal patterns) can be monitored for inference [12]. While state-space models have been used to monitor influenza in humans [13–15] as well as and for herd-management decisions [16–19], it has not yet been determined how useful these techniques are for monitoring endemic diseases.

The objectives of this study were to assess the performance of different statistical monitoring methods for endemic disease control programs, and to explore what impact of variation (noise) in the data had on the performance of these statistical monitoring methods. The simulation study was motivated by the Danish PRRSV monitoring program.

Two state-space models were chosen for this study based on their ability to monitor changes in different time-series components [11]. Five different statistical monitoring methods were evaluated for each model: three UPCA used in process-control monitoring [20], and two methods for monitoring changes based on the trend component of the time series.

## Materials and methods

All methods described in this section were implemented using R version 3.1.1 [21].

### Data

Laboratory submission data stored in the National Veterinary Institute–Technical University of Denmark (DTU Vet) information management system and in the Laboratory for Swine Diseases–SEGES Pig Research Centre (VSP-SEGES) were used to determine the weekly PRRS sero-prevalence in Danish swine herds between January 2007 and December 2014(418 weeks in total). The weekly PRRS sero-prevalence was calculated using the same method described in a previous study [7]. A total of 51,639 laboratory submissions from 5,095 Danish swine herds were included. The average between-herd PRRS sero-prevalence was 0.24 (minimum = 0, maximum = 0.38) and the median number of herds tested for PRRS was 122 (minimum = 8, maximum = 191) per week.

### Simulation study

A baseline scenario for sero-prevalence was defined based on the method described by Lopes Antunes et al. [7], where the number of positive herds per week was derived from a binomial distribution with probability (*p*) and sample size (*n*) equal to the number of Danish herds tested for PRRS in a given week. The data is publicly available at the following link: https://figshare.com/s/8760d1be0d738e57292b (DOI: 10.6084/m9.figshare.4272260).The weekly sero-prevalence was calculated as the simulated number of sero-positive herds divided by the total number of herds tested per week.

**Fig 1:**
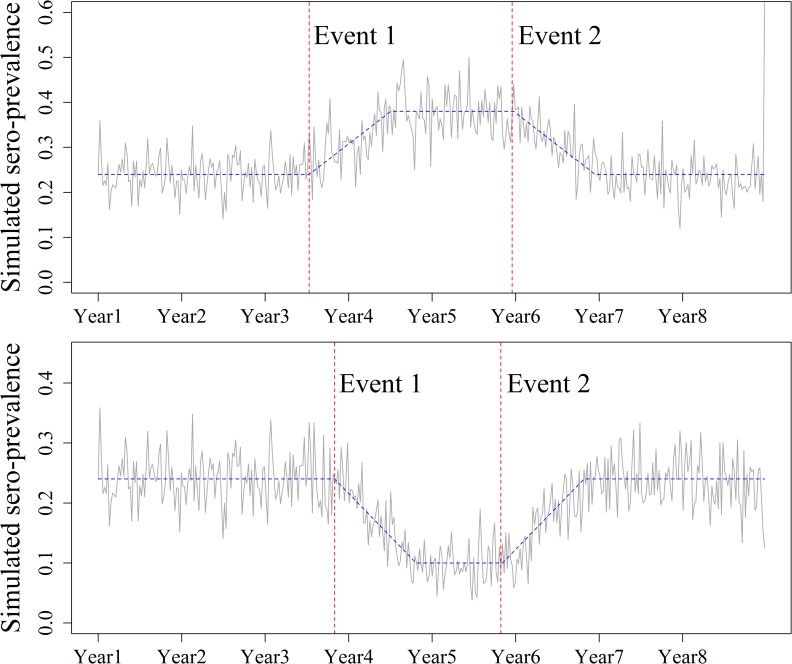
Simulated scenarios representing endemic disease monitoring. The between-herd weekly sero-prevalence was simulated using a binomial distribution based on the Danish herds tested for PRRSV during the corresponding week. An initial sero-prevalence of 0.24 was maintained for at least 104 weeks. This was followed by either an increase to 0.38 or a decrease to 0.10 over 52 weeks in two different events. The different statistical monitoring methods were evaluated for each event.

There was a constant initial sero-prevalence of 0.24 for the first 104 weeks of all simulated scenarios, corresponding to the average PRRS sero-prevalence observed in Danish herds in the diagnostic laboratory data from 2007 to 2014 (Fig 1). In Scenario A, this period was followed by an increase in the weekly sero-prevalence (Event 1), a constant level, and then a decrease (second event). Scenario B consisted of a decrease in the sero-prevalence (Event 1) followed by a constant level, then an increase during the subsequent weeks (Event 2). Each scenario was simulated with changes in the weekly sero-prevalence, including gradual increases to 0.33 and 0.38 (for Scenario A) and gradual decreases to 0.15 and 0.10 (for Scenario B) over 52 and 104 weeks. Different combinations and durations of events (increases/decreases in sero-prevalence) were tested for each scenario, resulting in a total of 16 simulated scenarios (Table 1). Event 1 of each scenario was started at a random time between weeks 104 and 156, and Event 2 was started after a random interval of between 52 and 104 weeks following the end of Event 1.

**Table 1:** Description of the 16 simulated scenarios representing changes in endemic diseases.

		Event 1		Event 2	
	Initial sero-prevalence	Sero-prevalence achieved at the end of the event	Duration of the event (weeks)	Sero-prevalence achieved at the end of the event	Duration of the event (weeks)
**Scenario A**	0.24	0.33	52	0.24	52
			52		104
			104		52
			104		104
		0.38	52		52
			52		104
			104		52
			104		104
**Scenario B**	0.24	0.15	52	0.24	52
			52		104
			104		52
			104		104
		0.10	52		52
			52		104
			104		52
			104		104

An initial constant sero-prevalence of 0.24 was simulated over 104 weeks. This was followed by an increase in sero-prevalence to 0.33 or 0.38 (Scenario A) or a decrease to 0.15 and 0.10 (Scenario B) over 52 and 104 weeks (Event 1). Event 1 was followed by a constant level of sero-prevalence, then by a second event (Event 2), corresponding to a decrease (Scenario A) or an increase (Scenario B) to the initial value of 0.24 over 52 and 104 weeks. Different combinations of event durations and changes in the sero-prevalence were tested, resulting in a total of 16 scenarios.

### Modeling

A Dynamic Linear Model (DLM) and a Dynamic Generalized Linear Model (DGLM), both with a linear growth component as described previously [11], were used to model the simulated data.

The general objective of state-space models is to estimate an underlying parameter vector from observed data (θ) combined with any prior information available at time 0 (D_0_), *i*.*e*. before an observation is made. The estimated parameter vector is updated each time there is a new observation (e.g. of the PRRS sero-prevalence). Specifically, the distribution of θ_*t*_ conditional on D_t_ (θ_*t*_|D_t_) is estimated for each time step *t*. These models can be used to estimate a one-step forecast of the mean, allowing for a comparison between observed and forecasted values.

Briefly, the DLM is represented by a set of two equations, defined as the observation equation (Eq 1) and the system equation (Eq 2).

$$Y_{t}=F^{\prime }\theta_{t}+v_{t},v_{t}∼N(0,V_{t})$$ (1)

$$\theta_{t}=G\theta_{t-1}+w_{t},w_{t}∼N(0,W_{t})$$ (2)

Where *Y*_*t*_ was the observed sero-prevalence for week *t*, and *V*_*t*_ and *W*_*t*_ are referred to as the observational variance and system variance, respectively. In our study, the observational variance was adjusted for the number of submissions in a given week (see Eq 5 below). The transposed design matrix *(****F’****)* had the following structure:

$$F^{\prime }=[\begin{array}{cc} 1 & 0 \end{array}]$$ (3)

Eq 2 describes the evolution of θ from time *t*-1 to *t*. The system matrix *(****G****)* for a local linear trend model is given as:

$$G=\left[\begin{array}{cc} 1 & 1\\ 0 & 1 \end{array}\right]$$ (4)

The linear trend component enabled us to include a time-varying slope (or local linear trend), allowing the system to adapt to a potential positive or negative trend for each *t*. Assuming that the PRRS sero-prevalence was not auto-correlated over time, the observational variance was defined as:

$$V_{t}=\frac{Y_{t-1}\;(1-Y_{t-1})}{n_{t}}$$ (5)

where n_t_ was the number of herds tested for PRRS that week.

Unlike the DLM, the DGLM was based on a binomial distribution. The observation equation (Eq 6) for the DGLM was defined as:

$$p_{t}=F_{t}^{\prime }{ϴ}_{t}$$ (6)

For both DLM and DGLM, the variance-covariance matrix (*W*_*t*_) describes the evolution of variance and covariance of each parameter for each time step. Rather than estimating *W*_*t*_, the system variance was modeled using a discount factor (δ), as previously described by [22] and [17].

### State-space model initialization and discount factors

Reference analysis was used to estimate the initial parameters *D*_0_∼[*m*_0_, *C*_0_] as described by West and Harrison [11].

The discount factors (δ) were defined using the method described by Kristensen [23], and were selected in order to optimize the performance of the model forecasts (i.e. minimizing the normalized forecast errors $e_{t}^{norm}$). The DLM and the DGLM models were run for 418 weeks with a constant simulated sero-prevalence of 0.24, using different δ-values ranging from 0.1 up to 1 in increments of 0.01. The δ-value that minimized the sum of the squared normalized forecast errors was chosen for the analysis. For both models, the forecast errors were normalized with respect to the forecast variance Q, such that $e_{t}^{norm}=e_{t}/\sqrt{Q_{t}}$.

### Monitoring methods

#### Univariate process control algorithms (UPCA)

Three monitoring methods were used to generate alarms: the Shewhart Control Chart, Tabular Cumulative Sums, and V-Mask [20]. These methods are useful when only small changes are expected in the data [20].

The Shewhart Control Chart and Tabular Cumulative Sums were applied to the normalized forecast errors, whereas the V-Mask was applied to simple cumulative sums of the normalized forecast errors. The first 104 weeks of data were used as a “burn-in” period for the models and the alarms were generated from the third year onwards (>108 weeks) when the simulated events started.

The fixed upper and lower control limits (*UCL* and *LCL*) required for the Shewhart Control Chart to generate alarms in a given week were calculated based on the following equations [20]:

$${UCL(f)}_{t}=\mu_{t}+L\;\sigma_{t}$$ (7)

$${LCL(f)}_{t}=\mu_{t}-L\;\sigma_{t}$$ (8)

where *μ*_*t*_ is the center line (*μ*_*t*_ = 0), *L* is the selected number of standard deviations and *σ*_*t*_ is the standard deviation of the normalized forecast errors from *t*>104.

The Tabular Cumulative Sums for week *t* were calculated as described by Montgomery [20]. This method accumulates derivations from *T*_0_ (target value) that are above the target with one statistic *C*^+^, and below the target with another statistic *C*^−^. The *C*^+^ and *C*^−^ for a given week (*t*) were calculated as:

$$C_{t}^{+}=max\{0,\;e_{t}^{norm}-(T_{0}+K)+C_{t-1}^{+}\}$$ (9)

$$C_{t}^{-}=mix\{0,\;(T_{0}-K)\;e_{t}^{norm}+C_{t-1}^{-}\}$$ (10)

where *T*_0_ = 0 and *K* is the reference value expressed as *K* = (1 * *σ*_*t*_)/2. Alarms were raised if $C_{t}^{+}$ or $C_{t}^{-}$ exceeded a threshold *H* (expressed in terms of the standard deviation) in a given week *t*. The starting values of $C_{0}^{+}$ and $C_{0}^{-}$ were defined as zero.

The V-Mask was applied to successive values of the cumulative sum of normalized forecast errors, which was calculated as follows [20]:

$${\mathrm{cumulative}\;\mathrm{sum}}_{\;t}=\sum_{j=1}^{i}e_{t}^{norm}$$ (11)

**Fig 2:**
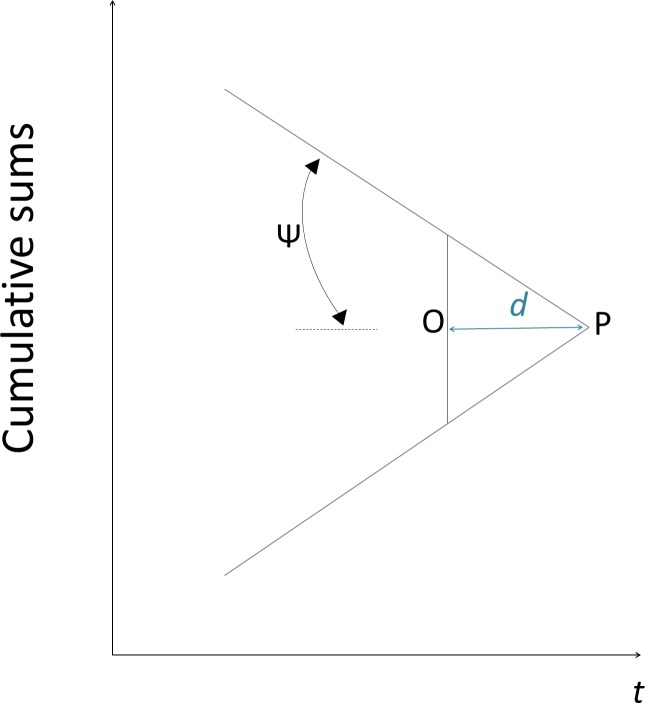
V-Mask description and application. The point O is positioned on the cumulative sum for each time *t*, and the line OP defines the lead distance d of the V-mask (a) as expressed using horizontal plotting time steps and it is applied to the cumulative sum (b).

The V-Mask is defined by the lead distance *d* and the angle *Ψ*, which were equivalent to the cumulative sum as described by Montgomery [20] (Fig 2). The point O of the V-Mask was directly applied to each value of the cumulative sum_*t*_ with the line OP parallel to the horizontal axis. The V-Mask was applied to each new point on the cumulative sum chart and the arms extended backwards towards the origin. If all the cumulative sums in previous time steps were within the two arms of the V-Mask, the process was considered to be ‘in-control’; if any of the cumulative sums lay outside of the arms, the process was considered ‘out-of-control’ and an alarm was given. The value of the cumulative sum_*t*_ was reset to zero each time an alarm was given.

#### Calibration

In order to calibrate the process control algorithms, the generalized DLM and DGLM were applied to 418 weeks of simulated data with a constant sero-prevalence of 0.24. The process control algorithms were calibrated for a false alarm rate of 1% when applied to the weekly $e_{t}^{norm}$ (excluding the first 104 weeks, which represented the “burn-in” period of both models). The Shewhart Control Chart was calibrated with *L* ranging from 1 to 4 standard deviations of the $e_{t}^{norm}$, and *μ*_*t*_ was defined as zero. For the Tabular Cumulative Sums, values of *H* ranging from 1 to 4 standard deviations of the $e_{t}^{norm}$ were tested. This process was simulated 2,000 times for each parameter of the algorithm during calibration, and the median value of the false alarm rate was used as the summary statistic for evaluation.

Montgomery [20] suggested using *Ψ* = tan^−1^(*K*) and *d* = H/K in order for the V-Mask to be comparable to the Tabular Cumulative Sums. For this reason, these values were adopted for the implementation of the V-Mask in this study.

#### Monitoring the time-series trend

**Fig 3:**
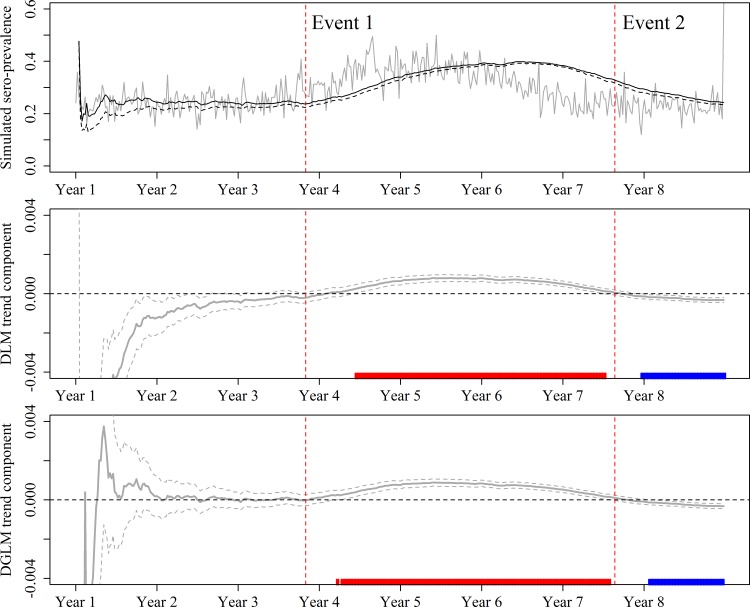
The results show the simulated weekly sero-prevalence and the filtered mean obtained from the DLM (black dashed line) and DGLM (solid black line), and the corresponding DLM and DGLM trend component. The rugs indicate where the trend component was significantly above (red) or below (blue) zero.

For both the DLM and DGLM, the trend was extracted from the θ vector for each time step *t*. The variance of the trend parameter was calculated from the variance-covariance matrix for the posterior distribution, as previously described [11]. This variance was used to calculate 99% confidence intervals (CI) (Fig 3). Alarms were generated based on the trend when significant differences above and below zero were found according to the 99% CI. In addition, a second method was used to generate alarms when the absolute values of the trend component changed the sign from positive to negative and vice versa (Trend Sign).

### Performance assessment

The performance was also assessed using the method proposed by Lopes Antunes et al [7]. The cumulative sensitivity (CumSe) was calculated as:

$${CumSe}_{i}=\frac{\sum_{j=1}^{i}x_{j}}{N_{iter}}$$ (12)

where *x*_*j*_ is the number of iterations in which an alarm was given *j* weeks after an event started, and *N*_*iter*_ is the total number of iterations. An increase in the sero-prevalence was considered to have been detected if an alarm was generated for each week *i* after the event was started (*i* ≥ 0).

### Convergence

A total of 10,000 iterations were simulated, with an initially constant sero-prevalence of 0.24 followed by a steady decrease to 0.15 over a period of 52 weeks. The decrease was randomly started between weeks 104 and 156. The number of iterations required to reach a stable detection time was determined visually using a plot of the variance in time to generate an alarm. This was done for each of the five statistical monitoring methods based on both types of models after the event was started with a stepwise increase of 100 iterations. Stable variance was observed after 2,000 iterations, therefore all simulated scenarios were run using this number of iterations.

### Assessing the impact of noise in the data on the performance of detection methods

**Fig 4:**
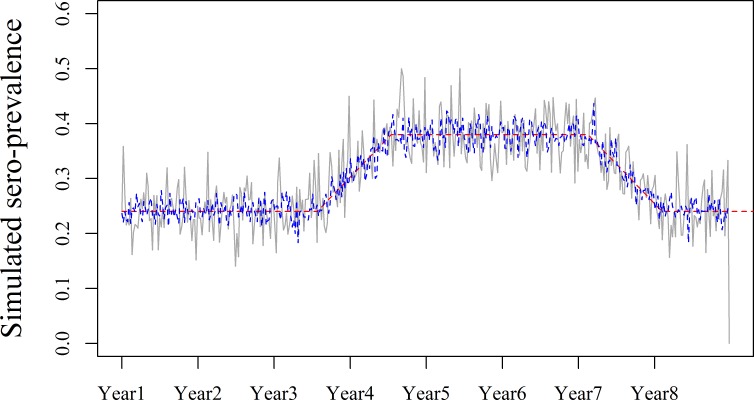
Simulated sero-prevalence representing endemic disease monitoring. The weekly sero-prevalence was simulated using a binomial distribution based on the Danish herds tested for PRRSV during the corresponding week (grey line), and with five times the average number of Danish herds (*n* = 600) tested for PRRSV (blue line). The red straight lines indicate the actual values of the simulated sero-prevalence.

In order to assess the impact of noise in the data, the simulation study was repeated with *n* fixed at 600 herds tested per week. This value corresponds to a five-fold increase in the average number of Danish swine herds tested for PRRSV per week between 2007 and 2014, and it reduced the variation in the baseline (Fig 4).

## Results

### Parameters used for calibration

The selected values used to define a 1% false alarm rate for the UPCA based on the DLM model corresponded to *L* = 2.6 for the Shewhart Control Chart, *H* = 6 and *K* = 6 for the Tabular Cumulative Sums, and a distance of 2 units for the V-Mask. For the DGLM model, the values corresponded to *L* = 2.5, *H* = 16, *K* = 5, and a distance of 3.2 units. These parameters were recalibrated to maintain a 1% false alarm rate when the number of herds tested per week was increased to 600, in order to simulate the baseline. The DLM model used parameters of *L* = 2.3 for the Shewhart Control Chart, *H* = 1.8 and *K* = 1 for the Tabular Cumulative Sums and a distance of 1.8 units for the V-Mask for a constant number of herds tested. For the DGLM model, these parameters were defined as *L* = 2.2, *H* = 11, *K* = 6 and a distance equal to 1.07 units.

A discount factor δ = 0.99 was used to define the system variance for the DLM and the DGLM.

### Statistical monitoring methods based on the DLM

The number of weeks needed to identify 50% of all iterations simulated (CumSe = 50%) for each event is given in Table 2. A CumSe = 50% was achieved most rapidly by the Trend Sign, followed by the V-Mask for Event 1 of Scenario A based on the DLM. For Event 2, the fastest CumSe = 50% was achieved using the V-Mask and Shewhart Control Chart. Using the Trend Sign to monitor the changes, we noted an increase in the number of weeks needed to achieve CumSe = 50% when comparing Event 1 and Event 2. As an example: for Event 1, 37 weeks were required to detect an increase in sero-prevalence from 0.24 to 0.38 over a period of 104 weeks based on 99% CI, and 2 weeks were required for the same increase and time period based on the Trend Sign. The same CumSe was achieved 74 and 59 weeks after the start Event 2 for the 99% CI and the Trend Sign, respectively. Furthermore, the Tabular Cumulative Sums detected changes in Event 1 of Scenario A more quickly than Event 2, with the exception of scenarios where changes occurred over 104 weeks. The main differences found when comparing scenarios A and B (Table 2) were: the Tabular Cumulative Sums was able to achieve a CumSe = 50% more quickly Event 2 of Scenario B than Scenario A; the Shewhart Control Chart achieved CumSe = 50% faster during Event 1 of Scenario B, and this value could not be achieved for Event 2 (expressed as NA in Table 2); the V-Mask quickly detected changes in Event 2 for Scenario B. Moreover, the 99% CI and the Trend Sign had similar results in both scenarios.

**Table 2:** Number of weeks needed to achieve a CumSe = 50% for the different statistical monitoring methods based on the DLM model.

		Event 1						Event 2					
	Sero-prevalence achieved	Duration (weeks)	Shewhart Control Chart^1^	Tabular Cumulative Sums^1^	V-Mask^2^	99% CI^3^	Trend sign^3^	Duration (weeks)	Shewhart Control Chart	Tabular Cumulative Sums	V-Mask	99% CI	Trend sign
**Scenario A**	0.33	52	119	27	18	34	2	52	31	52	18	121	93
	0.33	52	123	27	17	34	3	104	13	32	9	93	68
	0.33	104	146	44	20	50	1	52	30	52	18	113	89
	0.33	104	131	49	19	48	2	104	13	33	10	82	58
	0.38	52	121	19	13	27	2	104	26	43	16	109	93
	0.38	52	123	19	13	22	1	52	6	17	6	84	69
	0.38	104	158	33	18	39	1	104	25	43	16	103	89
	0.38	104	144	38	18	37	2	104	6	18	6	74	59
**Scenario B**	0.15	52	25	42	14	30	0	52	193	23	17	111	90
	0.15	52	25	42	13	30	0	104	NA	1	6	83	62
	0.15	104	35	70	18	46	0	52	NA	23	17	106	88
	0.15	104	39	75	18	43	0	104	NA	1	8	73	52
	0.10	52	19	29	10	23	0	52	NA	8	11	99	89
	0.10	52	19	28	10	23	0	104	NA	0	2	70	57
	0.10	104	28	51	14	35	0	52	NA	7	10	98	88
	0.10	104	32	58	16	33	0	104	NA	0	2	62	49

NA indicates that a CumSe = 50% was not achieved by the monitoring method.

^1^ Statistical monitoring methods applied to normalized forecast errors.

^2^Statistical monitoring methods applied to the simple cumulative sum of normalized forecast errors.

^3^ Statistical monitoring methods applied to the trend component.

Table 3 shows the CumSe_52_ (CumSe achieved 52 weeks after the event started) for the different statistical monitoring methods based on the DLM, indicating the likelihood of detecting the simulated events in the baseline for each method. For Scenario A, higher CumSe_52_ was achieved by the trend-based methods (99% CI and Trend Sign) and the V-Mask for Event 1. For Event 2, the Shewhart Control Chart and the V-Mask had higher CumSe_52_, and the trend-based methods were the worst performing (CumSe_52_≤0.3). When comparing scenarios A and B, the major differences were seen for the Shewhart Control Chart, corresponding to a better performance (higher CumSe_52_) for Event 1and a poorer performance for Event 2 of Scenario B. The other statistical monitoring methods presented similar results in both scenarios.

**Table 3:** CumSe achieved 52 weeks after the events were started for the different statistical monitoring methods based on the DLM model.

		Event 1						Event 2					
	Sero-prevalence achieved	Duration (weeks)	Shewhart Control Chart^1^	Tabular Cumulative Sums^1^	V-Mask^2^	99% CI^3^	Trend sign^3^	Duration (weeks)	Shewhart Control Chart	Tabular Cumulative Sums	V-Mask	99% CI	Trend sign
**Scenario A**	0.33	52	0.13	0.95	0.89	1.00	1.00	52	0.83	0.51	0.92	0.00	0.00
	0.33	52	0.11	0.94	0.87	1.00	1.00	104	0.98	0.70	0.96	0.00	0.12
	0.33	104	0.22	0.61	0.80	0.60	1.00	52	0.81	0.51	0.91	0.00	0.00
	0.33	104	0.24	0.52	0.81	0.65	1.00	104	0.98	0.68	0.96	0.01	0.37
	0.38	52	0.08	1.00	0.96	1.00	1.00	52	0.91	0.66	0.96	0.00	0.00
	0.38	52	0.07	1.00	0.96	1.00	1.00	104	1.00	0.93	0.99	0.00	0.08
	0.38	104	0.15	0.81	0.84	0.98	1.00	52	0.92	0.68	0.96	0.00	0.00
	0.38	104	0.18	0.70	0.82	0.97	1.00	104	1.00	0.91	1.00	0.04	0.33
**Scenario B**	0.15	52	0.94	0.66	0.95	1.00	1.00	52	0.14	0.93	0.90	0.00	0.00
	0.15	52	0.94	0.66	0.97	1.00	1.00	104	0.05	1.00	0.94	0.00	0.26
	0.15	104	0.71	0.37	0.86	0.74	1.00	52	0.16	0.93	0.91	0.00	0.00
	0.15	104	0.63	0.36	0.84	0.77	1.00	104	0.07	1.00	0.94	0.04	0.50
	0.10	52	1.00	0.93	0.99	1.00	1.00	52	0.08	1.00	0.96	0.01	0.01
	0.10	52	1.00	0.93	0.99	1.00	1.00	104	0.04	1.00	0.99	0.06	0.37
	0.10	104	0.84	0.52	0.94	1.00	1.00	52	0.17	1.00	0.97	0.00	0.00
	0.10	104	0.77	0.46	0.89	0.99	1.00	104	0.05	1.00	0.98	0.27	0.58

^1^ Statistical monitoring methods applied to normalized forecast errors.

^2^ Statistical monitoring methods applied to the simple cumulative sum of normalized forecast errors.

^3^ Statistical monitoring methods applied to the trend component.

### Comparing the results from both models

Results revealed that the statistical monitoring methods required more time to achieve CumSe = 50% when applied to DGLM (Table 4) compared to DLM (Table 2), with the exception of monitoring the Trend Sign in Event 1 (Scenario A) and the V-Mask in Event 1 (Scenario B). In these cases, CumSe = 50% was achieved at least twice as quickly for the DLM.

**Table 4:** Number of weeks needed to achieve a CumSe = 50% for the different statistical monitoring methods based on the DGLM model.

		Event 1						Event 2					
	Sero-prevalence achieved	Duration (weeks)	Shewhart Control Chart^1^	Tabular Cumulative Sums^1^	V-Mask^2^	99% CI^3^	Trend sign^3^	Duration (weeks)	Shewhart Control Chart	Tabular Cumulative Sums	V-Mask	99% CI	Trend sign
**Scenario A**	0.33	52	123	38	5	33	0	52	31	95	73	124	95
	0.33	52	127	37	6	35	0	104	11	72	71	96	69
	0.33	104	159	70	6	49	0	52	24	67	71	119	91
	0.33	104	157	214	3	47	0	104	12	81	68	83	58
	0.38	52	118	26	5	25	0	52	23	64	43	113	96
	0.38	52	120	25	5	21	0	104	5	38	88	86	71
	0.38	104	157	48	5	38	0	52	19	53	82	108	93
	0.38	104	152	95	3	36	0	104	5	41	77	75	59
**Scenario B**	0.15	52	52	128	5	32	1	52	162	27	9	101	82
	0.15	52	52	131	5	33	0	104	129	2	8	78	56
	0.15	104	93	172	5	48	0	52	141	24	8	97	80
	0.15	104	290	171	3	46	1	104	127	2	7	68	47
	0.10	52	36	117	5	25	1	52	164	17	10	84	76
	0.10	52	36	118	5	25	0	104	135	0	9	61	48
	0.10	104	65	161	5	37	0	52	153	13	8	82	74
	0.10	104	NA	154	3	34	0	104	133	0	7	52	39

NA indicates that a CumSe = 50% was not achieved by the monitoring method.

^1^Statistical monitoring methods applied to normalized forecast errors.

^2^ Statistical monitoring methods applied to the simple cumulative sum of normalized forecast errors.

^3^ Statistical monitoring methods applied to the trend component.

The trend-based methods produced identical results based on the DGLM (Table 5) and the DLM (Table 3). In general, these methods achieved the highest CumSe_52_ based on the DLM for all simulated scenarios.

**Table 5:** CumSe achieved 52 weeks after the events were started for the different statistical monitoring methods based on the DGLM model.

		Event 1						Event 2					
	Sero-prevalence achieved	Duration (weeks)	Shewhart Control Chart^1^	Tabular Cumulative Sums^1^	V-Mask^2^	99% CI^3^	Trend sign^3^	Duration (weeks)	Shewhart Control Chart	Tabular Cumulative Sums	V-Mask	99% CI	Trend sign
**Scenario A**	0.33	52	0.05	0.80	0.98	1.00	1.00	52	0.83	0.13	0.24	0.00	0.00
	0.33	52	0.04	0.78	0.98	1.00	1.00	104	0.99	0.39	0.22	0.00	0.09
	0.33	104	0.08	0.33	0.98	0.65	1.00	52	0.88	0.36	0.23	0.00	0.00
	0.33	104	0.07	0.12	1.00	0.70	1.00	104	0.99	0.37	0.26	0.00	0.36
	0.38	52	0.03	1.00	0.98	1.00	1.00	52	0.94	0.30	0.08	0.00	0.00
	0.38	52	0.03	0.99	0.98	1.00	1.00	104	1.00	0.63	0.09	0.00	0.05
	0.38	104	0.06	0.58	0.98	0.99	1.00	52	0.97	0.50	0.15	0.00	0.00
	0.38	104	0.05	0.22	1.00	1.00	1.00	104	1.00	0.60	0.15	0.02	0.32
**Scenario B**	0.15	52	0.51	0.03	0.98	1.00	1.00	52	0.03	0.90	0.98	0.00	0.00
	0.15	52	0.50	0.04	0.99	1.00	1.00	104	0.13	1.00	0.89	0.01	0.41
	0.15	104	0.26	0.03	0.99	0.69	1.00	52	0.20	0.94	0.96	0.00	0.00
	0.15	104	0.14	0.04	1.00	0.77	1.00	104	0.12	1.00	0.91	0.13	0.67
	0.10	52	0.82	0.04	0.98	1.00	1.00	52	0.01	0.99	0.98	0.00	0.01
	0.10	52	0.81	0.04	0.99	1.00	1.00	104	0.07	1.00	0.78	0.26	0.63
	0.10	104	0.38	0.03	0.99	1.00	1.00	52	0.11	0.99	0.96	0.00	0.02
	0.10	104	0.16	0.04	1.00	1.00	1.00	104	0.07	1.00	0.81	0.51	0.83

^1^Statistical monitoring methods applied to normalized forecast errors.

^2^Statistical monitoring methods applied to the simple cumulative sum of normalized forecast errors.

^3^ Statistical monitoring methods applied to the trend component.

### Impact of noise on the different detection methods

**Fig 5:**
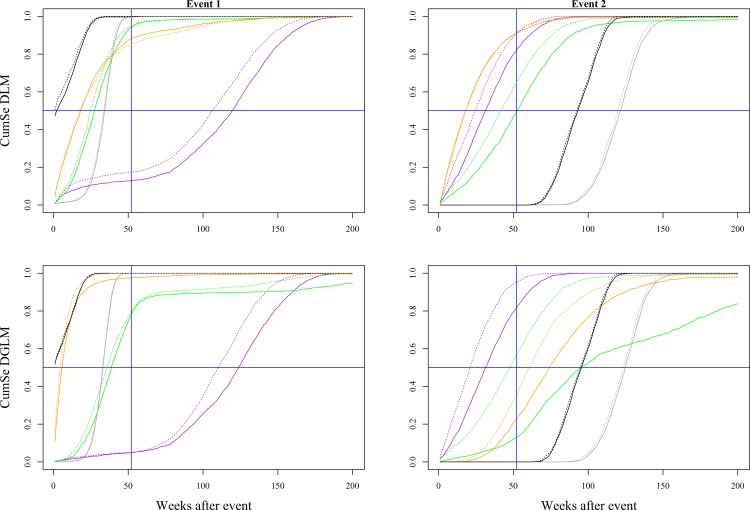
The impact of baseline variation on the cumulative sensitivity (CumSe) of the algorithms. The results are shown for Scenario A, corresponding to an increase in sero-prevalence from 0.24 to 0.33 over 52 weeks (Event 1), followed by a decrease from 0.33 to 0.24 over 52 weeks (Event 2). The CumSe of the Shewhart Control Chart (purple), Tabular Cumulative Sums (green), V-Mask (orange), 99% CI (grey) and Trend Sign (black) are shown based on the actual number of herds tested for PRRSV (straight lines) and on a fixed number (*n* = 600) of herds tested per week (dashed lines). The horizontal and vertical blue lines represent a CumSe = 50% and the CumSe achieved 52 weeks after the start of the event, respectively.

Reducing noise in the data (by increasing the sample size to 600 herds tested per week) resulted in higher CumSe for the statistical monitoring methods (Fig 5). The time required to achieve a CumSe = 1 was reduced by a factor ≥2 for the Shewhart Control Chart and Tabular Cumulative Sums. Similar results were found for the remaining 15 simulated scenarios (including Scenario B). The time required to achieve CumSe = 50% was reduced by 117 weeks for the Shewhart Control Chart for Event 1 of Scenario A, with an increase in sero-prevalence from 0.24 to 0.33 over 52 weeks based on the DLM. The Tabular Cumulative Sums achieved similar CumSe 8 weeks earlier based on the DLM than when based on the DGLM. The impact of baseline noise in the V-Mask and both trend-based methods had similar results, with only small differences (up to 2 weeks) in the time required to achieve CumSe = 50%.

## Discussion

We investigated the performance of different methods for detecting changes in endemic disease (sero-) prevalence. The study included: 1) univariate process control methods applied to residuals, and 2) monitoring changes in the trend component of the time series based on CI and absolute values. The Shewhart Control Chart detected increases in sero-prevalence better than decreases for both scenarios, whereas the opposite was observed for the Tabular Cumulative Sums. The trend-based methods were effective when detecting Event 1, but their performance was inferior when adapting to several consecutive events. The V-Mask seemed to be the method with the most consistent performance seemed to be. Additionally, the impact of noise in the baseline was more profound for the Shewhart Control Chart and Tabular Cumulative Sums, and lower for the V-Mask and the trend-based methods.

### Study design

This study was conducted based on sero-prevalence data from the Danish PRRS monitoring program. The different simulated scenarios were chosen to represent potential changes in sero-prevalence in the context of disease control programs, and were based on Danish pig production, where almost 40% of herds must follow rules concerning biosecurity, health control and transportation [9].

The approach used to simulate sero-prevalence was based on a binomial distribution defined by *n* and *p*. Both parameters have an effect on the variance of the binomial distribution, as higher values of *p* (up to 0.5) result in greater variance in the data obtained in each trial for a constant *n*, and lower values of *p* reduce the variance [24]. Event 1 of Scenario A and Event 2 of Scenario B represented an increase in sero-prevalence (*p*), resulting in greater variance of the data, which might have affected the detection rates presented in this study. However, higher values of *n* for the same value of *p* also have an impact on the variance of the simulated data, which facilitates the reduction of noise in the simulated time-series by defining *n* as five times the average number of herds tested.

A predefined false alarm rate of 1% was used for standardization, and to enable comparison between the different statistical monitoring methods. The value of 1% was chosen as a compromise between false alarms and maintaining confidence in the system.

### Results of the performance evaluation

Event 1 was started after 104 weeks in order to guarantee that the “burn-in” period of the model was sufficient for representative inferences to be made. From a practical point of view, false alarms can be generated, and true alarms can be masked thus reducing the sensitivity of the system for monitoring changes during this period.

As anticipated, larger changes in sero-prevalence were indicated earlier. These results are consistent with the expected performance of control charts [20].

The simulations showed that the Shewhart Control Chart was faster than the Tabular Cumulative Sums for detecting decreases in sero-prevalence. Conversely, the Tabular Cumulative Sums was faster at detecting increases. According to Montgomery [20], the Tabular Cumulative Sums is the recommended method for detecting gradual changes. However, the same author also mentioned that the Shewhart Control Chart might detect decreases earlier than the Tabular Cumulative Sum, as verified in this study. In addition, the variance in the simulated time-series was higher (due to a higher *p*) during Event 2 for Scenario B, which might explain the superior performance of the Tabular Cumulative Sums. Furthermore, the results for the trend component showed that both models needed time to adapt to Event 2 of both scenarios. It is possible that the models are forced to adapt to three consecutive stages of the sero-prevalence (“constant-event-constant”) prior to Event 2. This occurred because the system variance (modeled using a discount factor) was optimized for a constant level, resulting in slower model-trend changes for Event 2. As a consequence, the normalized forecast errors were higher and the Tabular Cumulative Sums generated alarms earlier, and as a result CumSe = 50% was achieved more quickly. The same argument can also be used to explain why the V-Mask attained a faster CumSe = 50% in Event 2 of Scenario B.

The V-Mask showed the most consistent results among the univariate methods in relation to the number of weeks required to achieve a CumSe = 50%. This can be explained by the greater flexibility of the V-Mask method compared to other univariate process control methods based on pre-defined control limits.

Regarding the trend-based methods, the Trend Sign was quicker at detecting changes than the 99% CI. However, it is possible that the instantaneous detection of Event 1 for both scenarios based on the Trend Sign might occur due to the variation (above and below zero) of the trend component. In this case, changes in the sign (from positive to negative and vice versa) might occur by chance.

### Impact of noise in the baseline

Decreasing the noise in the time-series resulted in higher CumSe for the Shewhart Control Chart and Tabular Cumulative Sums, whereas no important changes were found for the V-Mask or the trend-based methods. This shows the impact of variation in the time series and the importance of choosing the correct monitoring method. When the Shewhart Control Chart and Tabular Cumulative Sums were used, alarms were generated according to the intensity of noise in the data, regardless of whether they were applied to forecast errors or directly to the data. The superior performance of the Shewhart Control Chart may be due to the upper and lower control limits being defined based on data with less variation. Despite recalibrating to a 1% false alarm rate, the applied control limits were defined based on lower standard deviations, which contributed to the alarms being generated earlier. One possible explanation for the superior performance of the Tabular Cumulative Sums is that the noise in the simulated data was greater during the increase in sero-prevalence, thus increasing the chances of alarms being generated. There has also been previous reference to the impact of noise in the data on the Tabular Cumulative Sums [1,7].

Decomposing the time-series also offers a way to monitor the underlying trend usually masked by random noise in the data. Monitoring the trend component based on CI or target values provides a more stable pattern compared to monitoring the forecast errors.

### Perspectives

Choosing the correct methods for the prediction and determination of anomalies is critical for their effective detection [25]. Over the last decade, research has focused on the detection of (re-)emerging disease outbreaks [1–3]. Nevertheless, it is also important to follow up on implemented strategies in order to reduce and/or eliminate specific endemic diseases [6], and control and eradication programs play an important role within this context [26].

In this study, we showed that there is no robust method for all scenarios. Similar conclusions were drawn in previous studies on syndromic surveillance for (re)-emerging diseases [1,2,27,28], where the authors concluded that no single method was suitable for use with all outbreak signals. A surveillance system should be able to detect a variety of outbreaks with different characteristics [29,30]. This is important when the outbreak signature is unknown. The same challenges are extrapolated to the context of endemic diseases and eradication programs for monitoring changes in (sero-)prevalence.

The efficiency with which changes in prevalence were monitored varied among the different methods. Choosing one specific monitoring method is therefore challenging, and the objectives of the monitoring program and the performance of the statistical monitoring methods in different time patterns should be taken into account [31]. Furthermore, it is important to consider the objectives of the control program, the nature of the disease, political and economic factors, and the infrastructure of the country in which it will be implemented [32].

In this study, state-space models were used to monitor endemic disease and control programs using two distinctive monitoring approaches for the time-series components. The principles can also be applied to general modeling, and the monitoring and surveillance of (re-)emerging diseases in human and veterinary sciences. The need to monitor declining changes in the context of veterinary syndromic surveillance has previously been discussed [33]. This author referenced the importance of monitoring decreases in the number of submissions (such as a decrease in the compliance of farms with passive disease surveillance) and the need for detection and action in the context of active surveillance.

## Conclusions

Surveillance and monitoring systems are critical for the timely and effective control of infectious diseases. The different statistical monitoring methods used in this study performed differently in monitoring changes in disease sero-prevalence. In this context, choosing a single method is challenging, and the objectives of the monitoring program as well as the performance of the statistical monitoring methods in different time patterns should be taken into account. Furthermore, noise in the simulated baseline had an impact on the Shewhart Control Chart and the Tabular Cumulative Sums, whereas no substantial changes were found for the trend-based methods. Using the V-Mask or monitoring the trend component provided a consistent approach to monitoring changes in disease sero-prevalence.
